# Valosin containing protein (VCP/p97) expression in laryngeal squamous cell carcinoma: clinical evaluation and implications for targeted therapy

**DOI:** 10.3389/pore.2025.1612202

**Published:** 2025-11-21

**Authors:** Inga M. C. Seuthe, Hanna C. Hunsicker-Biederbeck, Markus Ruwe, Julius Knierer, Eric Ehrke-Schulz, Eren Erdogan, Sabine Eichhorn, Jonas J.-H. Park

**Affiliations:** 1 Department of Otorhinolaryngology, Head and Neck Surgery, Catholic Hospital Hagen, University of Witten/Herdecke, Hagen, Germany; 2 Institute for Pathology Hagen, Hagen, Germany; 3 Department of Human Medicine, Faculty of Health, Center of Biomedical Education and Research (ZBAF), Institute for Virology and Microbiology, University of Witten/Herdecke, Witten, Germany

**Keywords:** VCP, p97, LSCC, HNSCC, target therapy

## Abstract

**Purpose:**

Valosin-containing protein (VCP/p97) is a key regulator of proteostasis and cellular stress response and has been linked to tumor progression and poor prognosis in various malignant diseases. However, data on its role in laryngeal squamous cell carcinoma (LSCC) are lacking.

**Methods:**

In this retrospective single-center study, VCP/p97 protein expression was analyzed by immunohistochemistry in a cohort of 100 LSCC patients. Expression levels were semi-quantitatively assessed with H-Score, compared to normal tissue if possible and correlated with clinicopathological parameters. Survival analyses were evaluated by Cox regression.

**Results:**

VCP/p97 was expressed in all tumors. Most LSCC (77.0%) showed a uniform staining pattern. 46 of these tumors (59.7%) exhibited a staining intensity of 2–3. Among the tumors with a non-homogeneous staining pattern (n = 23), two tumors showed a predominance to lower staining (staining intensity 1). In 13 samples a comparison to normal epithelium was possible. In these samples, 9 (69.2%) samples showed higher VCP/p97 expression compared to the normal epithelium and 4 (30.8%) showed lower expression. VCP/p97 H-Score was not significantly associated with tumor stage, grade, lymph node status or patient survival.

**Conclusion:**

Although VCP/p97 expression is not prognostic in LSCC, its consistent expression may suggest a potential role as a molecular target. Further functional and translational studies are warranted to explore the therapeutic utility of VCP/p97 inhibition in LSCC.

## Introduction

Laryngeal squamous cell carcinoma (LSCC) is among the most prevalent malignancies of the head and neck and remains a significant clinical challenge. Smoking and excessive alcohol consumption are considered major risk factors for the development of LSCC [1]. In a study investigating the role of tobacco and alcohol consumption in LSCC, the multivariate odds ratio was 9.38 for smokers without alcohol abuse and 2.46 for non-smokers with excessive alcohol consumption (defined as more than 8 drinks per day) [2]. Other discussed risk factors include Human Papillomavirus (HPV), Epstein-Barr virus (EBV), asbestos, *Helicobacter pylori* infection, opium, and Agent Orange [1, 3].

Despite advances in surgical and non-surgical treatment modalities, 5-year survival rates have declined over recent decades [4]. Current prognostic tools are primarily based on tumor staging and histopathological grading, which often fail to accurately predict individual patient outcomes. Consequently, there is growing interest in identifying novel molecular markers that may refine prognostic assessment and offer potential therapeutic targets.

The Valosin containing protein (VCP, also known as p97) is a member of the family of AAA-ATPases and plays an important role in several cell functions. These include endoplasmic reticulum-associated degradation (ERAD), mitochondria-associated degradation (MAD) and the ubiquitin-proteasome system (UPS) [5]. It has already been associated with metastatic potential in cancer cells [6]. Furthermore, VCP/p97 inhibition can induce immunogenic cell death (ICD) in tumor cells [7, 8]. In addition, VCP/p97 is involved in the ubiquitin-dependent proteasomal degradation of IκBα, an inhibitor of NFκB [9]. NFκB is a mediator of the immune and inflammatory response as well as a protective factor against apoptosis [10].

VCP/p97 expression has already been reported in various solid tumors [11–20]. Furthermore, VCP/p97 has been reported to have prognostic significance in esophageal, gastric, prostate, pancreatic, follicular thyroid, gingival, breast, bronchial and HPV negative oropharyngeal carcinomas [11–20]. Thus, VCP/p97 represents a potential prognostic marker and an interesting therapeutic target in cancer therapy. Studies on the expression and prognostic significance of VCP/p97 in LSCC are lacking. To gain additional insights in this regard, a retrospective study on the expression and prognostic significance of VCP/p97 was conducted in a cohort of LSCC. To the best of our knowledge, this is the first study to investigate the expression and prognostic significance of VCP/p97 in LSCC.

## Methods

### Ethics statement

The study was approved by the local Ethics Committee (Ethics application number: S-234/2022) and was conducted according to the latest version of the Declaration of Helsinki.

### Subjects and material

100 samples of LSCC were analyzed for VCP/p97 expression. The samples were obtained during panendoscopy or during tumor resection. The clinical characteristics of patients are shown in Table 1. Tumor staging was determined according to the 8th edition of the American Joint Committee on Cancer Staging (AJCC) [21]. In most cases, the patients received surgical therapy. Follow-up time was defined as the time between the date of initial diagnosis and the last follow-up appointment or date of death. The median follow-up time was 49 months (range 0–142 months). The tissue was fixed in 4% buffered formalin and embedded in paraffin according to routine procedures. All sections were initially examined by the pathologist (M.R.) after hematoxylin-eosin staining to ensure that representative tumor tissues were selected for further investigation.

**TABLE 1:** Clinicopathological characteristics of the LSCC patients.

Characteristic	No. of patients	%	N (total)
Sex			100
Male	82	82.0	
Female	18	18.0	
Age			
Median	64		
Minimum	40		
Maximum	87		
T-stage			100
Tis	3	3.0	
1	35	35.0	
2	16	16.0	
3	23	23.0	
4	23	23.0	
N-stage			100
0	63	63.0	
1	7	7.0	
2	28	28.0	
3	2	2.0	
M-stage			100
0	96	96.0	
1	4	4.0	
X	0	0.0	
Grading			100
1	2	2.0	
2	51	51.0	
3	2	2.0	
4	40	40.0	
AJCC			100
0	3	3.0	
I	33	33.0	
II	10	10.0	
III	18	18.0	
IV	36	36.0	
Treatment			100
Surgery + RT/RCT/BRT	22	22.0	
RT/RCT/BRT alone	17	17.0	
Surgery alone	55	55.0	
No therapy	3	3.0	
Palliative therapy	3	3.0	
Regular smoking			100
+	85	85.0	
−	4	4.0	
Unknown	11	11.0	
Regular drinking			100
+	33	33.0	
−	16	16.0	
Unknown	51	51.0	

RT, Radiotherapy; RCT, Radiochemotherapy; BRT, Cetuximab-based bioradiotherapy.

### Immunohistochemistry for VCP/p97

**FIGURE 1:**
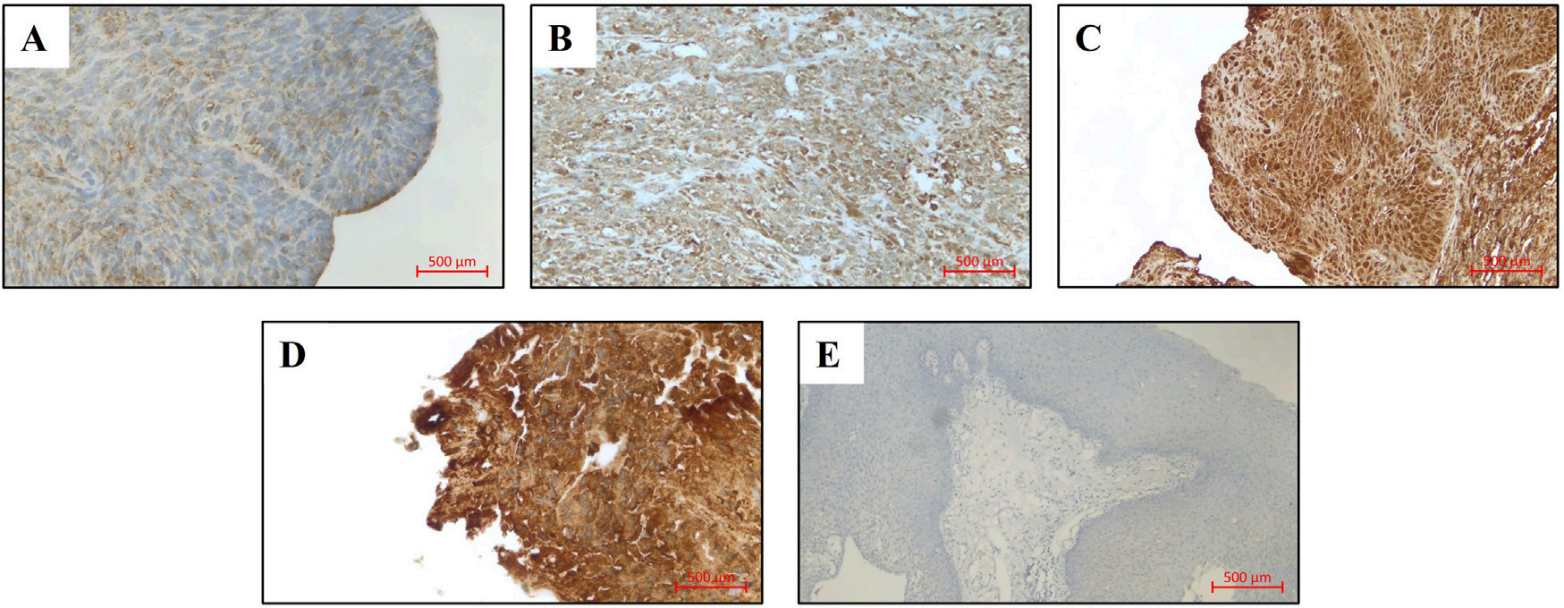
VCP/p97 expression in LSCC patients (original magnification: x200), **(A)** Expression level 0, **(B)** Expression level 1, **(C)** Expression level 2, **(D)** Expression level 3, **(E)** Negative control.

Histological slides were stained with the VENTANA BenchMark ULTRA Immunostainer (Roche). The immunohistochemical staining was performed using the biotin-free complex method. For detection of VCP/p97, a mouse anti-human monoclonal antibody (Santa Cruz Biotechnology, Cat. No. sc-57492) was applied at a dilution of 1:100. Staining was visualized using the ultraView Universal DAB Detection Kit (Roche). Known positive tissue for the antibody was co-stained as a positive control. In addition, in each staining run, the antibody dilution solution (Antibody Diluent, Roche) was applied to one slide instead of the primary antibody to serve as a negative control. Slides were classified by three authors (H.H.B., I.S., M.R.) in a blinded fashion without knowledge of the clinical-pathological data. We assessed the absolute staining intensity without comparison to non-carcinoma endothelial cells and categorized it as level 0 (no staining), level 1 (weak), level 2 (moderate) and level 3 (strong) (Figure 1). A H-Score was calculated for each sample by multiplying the percentage of tumor cells at each intensity by the corresponding intensity value using the following formula: H-Score= (0× % cells with intensity 0) + (1× % cells with intensity 1) + (2× % cells with intensity 2) + (3× % cells with intensity 3), yielding a final score ranging from 0 to 300. In histological sections where adjacent normal epithelium was available, a direct comparison was performed. The staining intensity was evaluated as more intense, equal, or less intense compared to the normal epithelium.

### Statistical analysis

For statistical analysis SPSS Base System (SPSS, version 29, Chicago, IL) was used. Normality of continuous variables was assessed using the Shapiro-Wilk test. Comparisons between groups were performed using the Mann-Whitney U test and the Kruskal-Wallis test. Overall survival (OS) and disease-free survival (DFS) were analyzed by Cox proportional hazards regression and reported as hazard ratios (HR) with 95% confidence intervals (CI). As an exploratory approach, the VCP/p97 H-score was additionally dichotomized at the median to generate Kaplan–Meier survival curves, and group differences were evaluated by the log-rank test. In testing a p-value <0.05 was considered statistically significant.

## Results

### VCP/p97 expression in LSCC

Immunohistochemical quantification revealed VCP/p97 H-scores ranging from 90 to 300, with a median of 200 (interquartile range 100–200). The Shapiro–Wilk test indicated that VCP/p97 H-scores were not normally distributed (p < 0.001). In the majority of LSCC samples (n = 77), a uniform staining pattern was observed across the tumor tissue. None of the samples were negative for VCP/p97 expression. When compared to normal epithelium (n = 13), 9 tumors (69.2%) showed a higher VCP/p97 staining intensity, while 4 tumors (30.8%) showed a lower staining intensity than the normal tissue.

### Correlation of VCP/p97 with clinical data

Among the clinicopathological parameters analyzed, only smoking status showed a significant association with the VCP/p97 H-score (Table 2).

**TABLE 2:** Association of VCP/p97 H-scores with clinicopathological parameters.

Characteristic	Category	N	H-score median (IQR/range)^a^	P-value^b^
T-stage	Tis	3	290 (120–300)	p = 0.203
	1	35	200 (100–200)	
	2	16	200 (127.5–290)	
	3	23	200 (100–200)	
	4	23	110 (100–200)	
N-stage	+	37	200 (100–267.5)	p = 0.306
	-	63	190 (100–200)	
M-stage	0	96	200 (100–200)	p = 0.688
	1	4	200 (100–300)	
Grading	1	2	190 (180–200)	p = 0.982
	2	53	200 (100–200)	
	3	40	200 (100–200)	
AJCC	0	3	290 (120–300)	p = 0.651
	I	33	200 (100–200)	
	II	10	200 (100–200)	
	III	18	190 (100–200)	
	IV	36	200 (100–200)	
Regular smoking	+	85	200 (100–200)	**p = 0.033**
	−	4	100 (100–180)	
Regular drinking	+	33	200 (100–200)	p = 0.673
	−	16	195 (100–200)	

IQR, Interquartile Range. Significant values were highlighted in bold.

^a^ Values are presented as median with interquartile range (IQR). For subgroups with small sample sizes (n ≤ 5), values are reported as median with range instead of IQR.

^b^ P-values were calculated using the Mann–Whitney U test (two groups) or the Kruskal–Wallis test (≥3 groups).

### Survival analysis

The 5-year survival rate for patients in the study population was 51.0%. The 5-year recurrence-free survival rate was 62.5%.

The VCP/p97 H-Score did not show a significant impact on either overall survival or disease-free survival over the full observation period. In the univariate Cox regression analysis, the VCP/p97 H-score was not significantly associated with overall survival (HR 0.999; 95% CI 0.995–1.003; p = 0.618) or disease-free survival (HR 1.003; 95% CI 0.997–1.008; p = 0.348). When dichotomized at a cut-off of 200 (Median), the VCP/p97 H-Score does not exhibit a significant impact on overall survival during the full observation period (21.5% (<200) vs. 33.0% (≥200), p = 0.350). There was also no significant association when looking at disease-free survival (67.2% (<200) vs. 60.7% (≥200), p = 0.772).

## Discussion

The retrospective study investigated the expression and prognostic significance of VCP/p97 in LSCC.

In various studies on solid tumors, increased VCP/p97 expression was shown [11–20]. Yamamoto et al. [15–21] and Tsujimoto et al. [14] compared the expression to non-cancerous endothelial cells. In the study by Meyer et al. [20] an absolute staining intensity was examined and assessed. An examination in comparison to normal epithelium was possible in 13 LSCC. 69.2% of these samples showed higher VCP/p97 expression than the normal tissue. The studies by Yamamoto et al. [12–17] and Tsujimoto et al. [11], that used the same evaluation, showed values between 58.8% and 78.8%. However, it is crucial to acknowledge that the limited number of LSCC samples that could be analyzed in our study is a limitation. In our LSCC cohort, 77 tumors showed a homogeneous staining pattern across the entire tumor tissue. Of these, 46 tumors (59.7%) exhibited a staining intensity of 2–3. This finding is in line with the oropharyngeal squamous cell carcinoma cohort reported by Meyer et al. [20], in which 52.8% of tumors demonstrated a staining intensity of 2–3. Among the tumors with a non-homogeneous staining pattern (n = 23), two tumors showed a predominance to lower staining intensity, in both cases corresponding to intensity 1.

Elevated VCP/p97 levels have been linked to lymph node metastasis in follicular thyroid, esophageal, gastric, pancreatic and breast carcinoma [13, 14, 16, 17, 19]. A possible explanation could be the involvement of VCP/p97 in the regulation of nuclear factor κB (NFκB) [6]. NFκB is a mediator of the immune and inflammatory response as well as a protective factor against apoptosis [10]. VCP/p97 is involved in the ubiquitin-dependent proteosomal degradation of IκBα, an inhibitor of NFκB [9]. For instance, a constant activation of NFκB, a reduced apoptosis after cell treatment with TNF-α and an increased metastatic potential were shown in VCP/p97 transfected osteosarcoma cells [6]. However, no association between VCP/p97 H-Score and lymph node metastasis was observed in our LSCC cohort. A significant correlation between high VCP/p97 expression and advanced T-stage has been demonstrated for gingival squamous cell carcinomas, gastric carcinomas, follicular thyroid carcinomas, and colorectal carcinomas [14–16, 18]. This was not found in our collective of LSCC. In LSCC VCP/p97 expression does not appear to be decisively involved in tumor invasion or metastasis development. However, it is important to note that multiple functions of VCP/p97 are co-regulated by various cofactors, such as FAF1, UFD1/NPL4, and HSP90 [22]. The adapter protein FAF1 has been shown to inhibit the activation of IκB kinase by interfering with kinase complex assembly. This prevents the phosphorylation of IκB and suppresses NFκB [23]. Downregulation of the adapter protein FAF1 is also expected to lead to an increase in NFκB activity. In oropharyngeal carcinomas it has been shown that FAF1 gene expression is associated with the occurrence of locoregional recurrence [24]. For the cofactor complex UFD1/NPL4, upregulation of NPL4 is known to promote cell proliferation in bladder cancer, while suppression of NPL4 reduces cell proliferation [25]. In oral lichen planus (OLP) and oral squamous cell carcinoma (OSCC), HSP90 expression is significantly increased in OSCC compared to OLP. HSP90 is also a known as an independent prognostic factor for shorter overall survival in patients with OSCC [26]. It is therefore possible that it may not be the overexpression of the ATPase VCP/p97 that plays the decisive role in tumor invasion in LSCC, it may be the expression of the adapter proteins. However, further investigations are necessary. In LSCC VCP/p97 H-Score showed no significant association to overall and disease-free survival. Studies investigating prostate, esophageal, follicular thyroid, gastric, non-small cell lung, pancreatic ductal, breast and colorectal carcinoma demonstrated significantly improved overall and disease-free survival rates in patients with low VCP/p97 expression [11–18]. Furthermore, significantly enhanced overall survival rates was observed in cases of gingival squamous cell carcinoma and breast carcinoma in patients with low VCP/p97 expression [15, 19]. In HPV negative oropharyngeal carcinomas, a significantly improved disease-free survival was demonstrated with high VCP/p97 expression [20]. This observation suggests that the role of VCP/p97 in tumor biology may be context- or tumor-type specific. Nevertheless, the findings of our study indicate that VCP/p97 is not a significant prognostic factor in LSCC.

In our LSCC cohort, regular smokers showed a significantly higher VCP/p97 H-score compared with nonsmokers (p = 0.033). This finding suggests that tobacco consumption may be associated with increased expression of VCP/p97. This is consistent with investigations in non-small cell lung carcinoma, in which a significantly higher expression of VCP/p97 was reported in patients with a positive smoking status [12]. Experimental studies further show that cigarette smoke induces proteostasis stress, aggresome formation, and autophagy disturbances in epithelial cells and recruits VCP/p97 more extensively into protein quality control, supporting the biological plausibility of this association [27, 28], These findings suggest that chronic tobacco exposure may trigger an adaptive upregulation of VCP/p97 to compensate for smoke-induced protein misfolding and ER stress.

However, interpretation of our results is limited by the unequal group sizes. While 85 patients were classified as smokers, the nonsmoker group comprised only four cases. Despite reaching statistical significance, the finding should therefore not be overinterpreted. The results should be regarded as exploratory and require confirmation in larger, more balanced cohorts.

If the association between tobacco consumption and VCP/p97 can be reproduced in further studies, this could have clinical relevance. For instance, there may be implications for biomarker-based therapeutic strategies, as smoking may influence VCP/p97 expression and thereby modulate the response to specific therapies.

The frequent expression of VCP/p97 in LSCC suggests that it may represent a potential therapeutic target. VCP/p97 is being investigated in cancer research due to the fact that many types of cancer appear to be particularly dependent on VCP/p97 and other proteostasis factors [29]. Several VCP/p97 inhibitors are available, including CB-5083, CB-5339, NMS-873 and DBeQanalogs. Furthermore, the inhibition of VCP/p97 is known to induce immunogenic cell death (ICD) in tumor cells [7, 8]. CB-5339 has already been tested in a Phase 1 trial in acute myeloid leukemia and myelodysplastic syndrome (https://clinicaltrials.gov, trial number NCT04402541). The results of the study have not yet been published. In bladder cancer using the RT112 mouse model, the combination of CB-5083 and radiotherapy resulted in significant enhanced tumor growth inhibition compared to radiotherapy alone or to the combination of radiotherapy plus mirin therapy [30]. In esophageal squamous cell carcinoma cell lines, the VCP/p97 inhibitor NMS-873 was shown to reduce cell proliferation in a dose-dependent manner. Moreover, the combination of radiotherapy and NMS-873 resulted in significant enhanced tumor cell death in esophageal carcinoma cells compared to radiotherapy alone [31]. Recently, Zhang et al. [32] developed several novel benzylquinazoline derivatives as potent VCP/p97 inhibitors with improved efficacy and reduced toxicity compared to established compounds. An experimental study to investigate the potential of VCP/p97 inhibitors as a therapeutic approach for the treatment of LSCC seems reasonable. As mentioned before, VCP/p97 was expressed not only in tumors but also in normal epithelium. In 69.2% of the samples exhibited higher tumor expression, while 30.8% showed lower levels. These results may suggest that VCP/p97 expression is generally maintained or even upregulated in tumor tissue, supporting its potential as a therapeutic target. However, the presence of VCP/p97 in normal epithelium indicates that systemic inhibition could also affect non-neoplastic tissues, raising concerns regarding potential on-target toxicities. To overcome this limitation, therapeutic strategies aiming at a more tumor-selective delivery of VCP/p97 inhibition might be required. One conceivable approach could be the use of adenoviral vectors engineered to express VCP/p97 inhibitors preferentially in tumor cells, thereby maximizing antitumor efficacy while minimizing damage to normal tissues. This underscores the need for future functional and preclinical studies to define the therapeutic window and safety profile of VCP/p97-targeted approaches. Further investigation of VCP/p97 inhibition in LSCC models is therefore warranted.

Several aspects of this study should be considered when interpreting the results. The investigation is based on a retrospective, single-center design, which may entail certain limitations regarding the generalizability of the findings. Moreover, expression analyses were performed using immunohistochemical methods which, although well established, allow only a semiquantitative assessment and do not provide direct insights into the functional activity of VCP/p97 or its potential interactions with regulatory co-factors. Nevertheless, the study offers initial indications of the relevance of VCP/p97 in LSCC and provides a basis for further investigations, particularly with respect to its therapeutic potential.

Our study demonstrates that VCP/p97 is consistently expressed in LSCC but lacks prognostic significance in this cohort. However, given its known involvement in proteostasis regulation, apoptotic resistance, and oncogenic signaling pathways, the frequent expression may support further evaluation of VCP/p97 as a molecular target in LSCC. Further functional studies and translational investigations are warranted to evaluate the therapeutic value of VCP/p97 inhibition in this malignancy.

## Data Availability

The raw data supporting the conclusions of this article will be made available by the authors, without undue reservation.
